# Kids Safe and Smokefree (KiSS) Multilevel Intervention to Reduce Child Tobacco Smoke Exposure: Long-Term Results of a Randomized Controlled Trial

**DOI:** 10.3390/ijerph15061239

**Published:** 2018-06-12

**Authors:** Stephen J. Lepore, Bradley N. Collins, Donna L. Coffman, Jonathan P. Winickoff, Uma S. Nair, Beth Moughan, Tyra Bryant-Stephens, Daniel Taylor, David Fleece, Melissa Godfrey

**Affiliations:** 1 Department of Social and Behavioral Sciences, College of Public Health, Temple University, 1301 Cecil B. Moore Ave, 9th Floor Ritter Annex, Philadelphia, PA 19122, USA; collinsb@temple.edu (B.N.C.); umanair@email.arizona.edu (U.S.N.); mgod521@temple.edu (M.G.); 2 Department of Epidemiology and Biostatistics, College of Public Health, Temple University, 1301 Cecil B. Moore Ave, Philadelphia, PA 19122, USA; dcoffman@temple.edu; 3 Massachusetts General Hospital Division of Pediatrics, 125 Nashua St, Suite 860, Boston, MA 02144, USA; jwinickoff@mgh.harvard.edu; 4 Health Promotion Sciences Department, 3950 S. Country Club Rd, Suite 300, PO Box: Abrams 300, Tucson, AZ 85714, USA; 5 Temple Pediatric Care, Temple University School of Medicine, 3509 N. Broad St, Philadelphia, PA 19140, USA; Beth.Moughan@tuhs.temple.edu (B.M.); David.Fleece@tuhs.temple.edu (D.F.); 6 Roberts Pediatric Clinical Research Building, Children’s Hospital of Philadelphia, 26 South St, 9th Floor, Philadelphia, PA 19146, USA; stephenst@email.chop.edu; 7 Department of Pediatrics, St. Christopher’s Hospital for Children, Front and Erie, Philadelphia, PA 19134, USA; drt23@drexel.edu

**Keywords:** secondhand smoke, pediatric, tobacco control, smoking cessation, intervention

## Abstract

Background: Pediatricians following clinical practice guidelines for tobacco intervention (“Ask, Advise, and Refer” [AAR]) can motivate parents to reduce child tobacco smoke exposure (TSE). However, brief clinic interventions are unable to provide the more intensive, evidence-based behavioral treatments that facilitate the knowledge, skills, and confidence that parents need to both reduce child TSE and quit smoking. We hypothesized that a multilevel treatment model integrating pediatric clinic-level AAR with individual-level, telephone counseling would promote greater long-term (12-month) child TSE reduction and parent smoking cessation than clinic-level AAR alone. Methods: Pediatricians were trained to implement AAR with parents during clinic visits and reminded via prompts embedded in electronic health records. Following AAR, parents were randomized to intervention (AAR + counseling) or nutrition education attention control (AAR + control). Child TSE and parent quit status were bioverified. Results: Participants (*n* = 327) were 83% female, 83% African American, and 79% below the poverty level. Child TSE (urine cotinine) declined significantly in both conditions from baseline to 12 months (*p* = 0.001), with no between-group differences. The intervention had a statistically significant effect on 12-month bioverified quit status (*p* = 0.029): those in the intervention group were 2.47 times more likely to quit smoking than those in the control. Child age was negatively associated with 12-month log-cotinine (*p* = 0.01), whereas nicotine dependence was positively associated with 12-month log-cotinine levels (*p* = 0.001) and negatively associated with bioverified quit status (*p* = 0.006). Conclusions: Pediatrician advice alone may be sufficient to increase parent protections of children from TSE. Integrating clinic-level intervention with more intensive individual-level smoking intervention is necessary to promote parent cessation.

## 1. Introduction

Tobacco smoke exposure (TSE) is a preventable cause of illnesses, disease, and death in children [1,2,3,4,5]. The primary source of child TSE is from a parent who smokes [6]. Young children are especially vulnerable to TSE and its adverse effects on health [7,8,9]. Urban, low-income, and minority children are at particularly high risk for TSE. One study found TSE in slightly over 70% of a predominantly African American sample of children from Detroit, with rates of TSE being inversely related to income and maternal education [10]. Biomarker (cotinine) data from a nationwide study showed that, during 2011–2012, an estimated 37.2% of non-Hispanic white children and 67.9% of non-Hispanic black children were exposed to tobacco smoke [11]. Despite significant declines in smoking among United States (US) adults in recent years [12], child TSE continues to be a significant public health threat, and interventions must reach low-income and predominantly minority urban families with a smoker to reach those children at highest risk of TSE.

Behavioral intervention strategies designed to eliminate child TSE generally focus on increasing parents’ efforts to protect children from TSE (e.g., establishing household smoking bans) or quit smoking. Both approaches tend, on average, to result in small improvements in quitting, protective behaviors, and child TSE reduction [13,14]. Addressing parent smoking and child TSE in communities that are at highest risk for child TSE is especially challenging. Compared with smokers of a higher socioeconomic status (SES), lower-SES smokers are less likely to report an intention to quit smoking [15], are less likely to use evidence-based aids to quit [16], and tend to be less successful in quitting [17,18]. Low SES also has been linked to a lower adoption of smoking bans in households with young children [19,20]. In terms of race, non-Hispanic black adults have more interest in quitting than non-Hispanic white adults, but are less likely to use evidence-based treatments or to successfully quit [21].

Previously, we reported on the theory, methods, and short-term (three-month, end of treatment; EOT) outcomes of our Kids Safe and Smokefree (KiSS) program, a child TSE reduction and parent cessation intervention that uses a multilevel intervention approach to deliver evidence-based interventions to low-income and minority parents who smoke [22,23]. Since the target population in KiSS faces considerable barriers to accessing evidence-based treatments, and confronts more challenges to quitting than the general population due to high levels of life stress [24,25,26,27], we hypothesized that a multilevel treatment model would be especially effective. This hypothesis was guided by a review of smoking cessation interventions in medical practices in which Kottke [28] concluded that, compared with single sources of information provided to a smoker in a single session, multiple sources of health information providing a greater number and longer duration of reinforcing sessions are related to cessation success. In addition, other reviews of smoking cessation intervention studies have concluded that more intensive cessation interventions result in substantially higher abstinence rates than minimal contact interventions [29]. Therefore, we reasoned that providing smoking parents with advice and education about the harms of child TSE and the benefits of protecting children from TSE from multiple message sources and in repeated doses would be effective for reducing child TSE in a high-risk population. Similarly, we anticipated that this approach would be effective at promoting smoking cessation in parents.

The design of the multilevel KiSS intervention was guided by ecological and associative learning theory, particularly social cognitive theory [30] and the behavioral ecological model [31]. Since smoking is a multi-determined behavior, the intervention addressed multiple levels of influence (e.g., psychological, environmental, social, and biological), which is also consistent with the ecological approach to addressing tobacco-related health disparities [32]. In the KiSS intervention, smoking parents were recruited from urban pediatric clinics serving low-income communities. Trial recruitment procedures were part of our broader clinic-level intervention (following “Ask, Advise, Refer” [AAR] best practice guidelines) [33] that all parents received from pediatric providers during their child’s medical visit. Consented parents were randomized to a telephone counseling intervention condition that reinforced clinic-level messaging and provided support, education, and cognitive-behavioral skills training to promote child TSE reduction and parent smoking cessation (AAR + counseling) or an attention control nutrition education condition (AAR + control).

Treatment fidelity analyses revealed high adherence among the clinicians (>80% of respondents recalled being encouraged to avoid smoking indoors, being advised about child TSE harms, and being advised about the benefits of reducing TSE) and the telephone counselors (≥90% protocol adherence among counselors) [22]. Analyses of parent-reported outcomes at EOT suggested that significantly more parents in the multilevel AAR + counseling condition than the AAR + control condition eliminated all of the sources of child TSE (45.8% versus 29.9%) and quit smoking (28.2% versus 8.2%) [22]. In addition, relative to the AAR + control condition, parents in the AAR + counseling condition at EOT were more likely to report home and car smoking restrictions, greater use of smoking urge management strategies, greater self-efficacy to protect children from TSE, and greater self-efficacy to quit smoking.

The purpose of this report is to update our parent-reported EOT outcomes with 12-month follow-up data, which includes bioverified outcomes of child exposure and parent quit status that were unavailable at EOT. First, we hypothesized that relative to children in the AAR + control arm, children in the AAR + counseling arm would have greater reductions in child TSE from baseline to 12-month follow-up, as measured by child urine cotinine, which is a biomarker for nicotine exposure. Second, we hypothesized that relative to parents in the AAR + control arm, parents in the AAR + counseling arm would have higher rates of bioverified quit status at 12-month follow-up. Third, when testing the first and second hypotheses, we included four non-program factors that could influence the risk of child TSE or parents’ ability to quit smoking: child age [11], the presence of other smokers in the home [34], parents’ level of nicotine dependence [35], and parents’ level of depressive symptoms [36].

## 2. Methods

### 2.1. Trial Overview

The KiSS trial used a randomized, two-group design with measurement at baseline, three-month (EOT), and 12-month follow-up. This report focuses on bioverified child TSE (urine cotinine levels) at baseline and 12-month follow-up, and parent quit status, which was bioverified by saliva cotinine at 12-month follow-up. All parents received pediatric clinic-level intervention (AAR) whether they were interested in the trial or not, as this was part of clinic quality improvement efforts to offer recommended best care for clients living with a parent who smokes. It also allowed the clinics to engage in meaningful use of their electronic health record (EHR) system [37]. After AAR, interested parents provided permission to the pediatric provider for the KiSS research staff to contact them by telephone to tell them more about the trial and screen them for eligibility. Eligible participants gave informed consent for inclusion before they participated in the trial. The trial was conducted in accordance with the Declaration of Helsinki, and the protocol was approved by the Temple University Institutional Review Board (protocol #20045). After consent, participants completed a structured interviewer-administered baseline assessment by phone. The interview was followed by a home visit to deliver some study materials and collect a child urine sample that would be used to assess nicotine exposure via urinary cotinine. On average, the time between the clinic visit and the telephone screen was five days, the time between the telephone screen and the baseline interview was four days, and the time between the telephone screen and the home visit was eight days. Participants were then randomized to a 12-week treatment in either: (a) an experimental group (AAR + counseling) that received print materials and up to five sessions of telephone counseling; or (b) an attention control group (AAR + control) that received print materials and telephone nutrition education. The trial was guided by CONSORT (Consolidated Standards of Reporting Trials) criteria [38].

### 2.2. Study Population

Eligible participants included tobacco smoking parents living in low-income, Philadelphia communities with a child under 11 years old who was exposed daily to tobacco smoke in the home. Participants were seen in our partnering pediatric clinics, which were part of three large hospital systems in Philadelphia. Additional inclusion criteria included being a daily smoker, >17 years old, and English speaking. Exclusion criteria included pregnancy, psychiatric diagnosis, non-nicotine drug dependence, daily consumption of > two alcoholic beverages. Participants were enrolled from September 2012 to June 2015. Projected sample size was calculated using power ≥.80 with 𝛼 = 0.05 and assuming ≤25% attrition.

### 2.3. Clinic-Level Intervention (“AAR” Pre-Randomization)

The clinic intervention emphasized pediatric providers’ adherence to AAR. To achieve objectives, investigators: (a) provided in-service training on following AAR steps, including child TSE advice to parents of exposed children and the provision of NRT (nicotine replacement therapy) prescriptions to interested parents; (b) embedded TSE assessment codes and a decision aid tool within the EHR that prompted simple, routine AAR adherence; (c) supplied providers with printed TSE and cessation resources; and (d) provided periodic feedback to clinic liaisons about providers’ AAR adherence. Clinics were detailed with posters, flyers, and brochures that had messages about TSE harms and AAR cues to action for pediatricians. Intervention steps included asking all of the parents if their child was exposed to tobacco smoke, advising parents of child TSE dangers and the benefits of child TSE protection, and providing self-help print materials (Environmental Protection Agency’s brochure, “Secondhand Smoke and the Health of Your Family”) and referral resources (Pennsylvania Quitline number, information on how to access free NRT). Providers faxed interested parents’ contact information to study staff. Clinician adherence was good [22].

### 2.4. Participant Assessment and Randomization Procedures

Trained and supervised research assistants conducted self-report assessments using structured telephone interviews. Quality control related to assessment was described in an early publication [22]. Eligible, consented participants completed baseline assessment interviews prior to randomization. Randomization used a permuted block design of varying lengths with two strata (hospital clinic site and race). Sealed, opaque envelopes organized by strata were created by the project biostatistician to conceal assignment information from research staff until immediately prior to intervention assignment. Assessment staff were blind to treatment assignment.

### 2.5. Telephone Counseling Interventions

#### 2.5.1. Counselor Training and Supervision

Intervention staff were trained master’s degree-level health counselors who participated in weekly supervision with a PhD-level clinical supervisor to review recorded telecounseling sessions against a treatment fidelity checklist to maintain ≥90% protocol fidelity. Details on training and fidelity maintenance can be found in an earlier publication [22].

#### 2.5.2. AAR with Telephone Counseling (Experimental Condition)

The experimental AAR + Counseling intervention included the clinic-level AAR plus up to five manualized telephone counseling sessions over 12 weeks. Prior to counseling, participants received an in-home program orientation in which they received a treatment binder, a schedule, and a copy of the Environmental Protection Agency brochure with cessation referral resources and NRT information to ensure its receipt, even if clinics did not provide these print materials. The timing, structure, and content of sessions were guided by Quitline best practices [39] and our previous intervention, Family Rules for Establishing Smoke-Free Homes (FRESH) [40]. Sessions used cognitive behavioral therapy strategies [41] (e.g., goal setting, self-monitoring, skills training) and social support to guide child TSE reduction efforts, as well as motivational interviewing techniques to facilitate a personalized cessation plan [42,43]. Telephone counselors were trained to provide: (a) support for smoking behavior change, (b) education on smoking urge management coping skills, (c) aid around goal setting and skill building to boost self-efficacy for child TSE protections and smoking cessation, and (d) guidance and navigation on how to acquire and properly use NRT.

#### 2.5.3. AAR with Telephone Nutrition Education (Attention Control)

The AAR + control group received AAR plus telephone-based nutrition education, which paralleled the attention delivered in the experimental arm. At the home visit orientation, staff provided the Environmental Protection Agency brochure to participants, cessation referral resources, and NRT information to ensure its receipt even if clinics had not provided these materials. Participants also received Sesame Street’s tool kit, “Food for Thought: Eating Well on a Budget” [44].

### 2.6. Measures

#### 2.6.1. Primary Treatment Outcome Variables

Child TSE was assessed via urine cotinine levels. Child urine was collected by parents at baseline and 12-month home visit assessment sessions, and stored in −80 °C freezer using biosafety-compliant, standardized collection and storage protocols. Cotinine assays were conducted at the Sports Medicine Research and Testing Laboratory (Salt Lake City, UT, USA) using a validated, sensitive, high-performance liquid chromatography-tandem high-resolution mass spectrometry method. The limit of quantitation was 0.1 ng/mL. Quality control was monitored by the analysis of blanks and a known standard at every 15th sample. Samples were labeled with a numerical code so that the lab conducting the assays was blind to participants’ identity and experimental condition. Cotinine remains the gold standard of biomarkers for assessing child TSE [45]. Urine cotinine is preferred over serum cotinine in child populations because it is less invasive. Parents’ self-reported seven-day point prevalence quit status and bioverification data obtained via saliva cotinine (Nicalert^TM^) and expired carbon monoxide (CO; for participants using NRT) were collected at the 12-month home visit assessment session.

#### 2.6.2. Covariates and Process Variables

Interviewers collected baseline participant demographics (e.g., age, race, education), smoking history (e.g., nicotine dependence measured by the Fagerström Test for Nicotine Dependence; FTND [46]), and psychosocial variables (e.g., depressive symptoms measured with the Center for Epidemiological Studies Depression Short Form) [47]. In addition, we assessed participants’ engagement in behaviors that were recommended to everyone during the in-clinic AAR intervention either by the provider or in the print materials (e.g., implement restrictions on smoking in the home, call the PA state quitline, acquire NRT). Parents’ 12-month adherence to recommendations was assessed through a self-report of: (a) the number of cigarettes smoked near the child by the parent and all other sources in the prior seven days; (b) implementation of *residential/car smoking restrictions* (0 = “no restrictions” versus 1 = “smoking restrictions enforced”); (c) calls to the PA state quitline (“yes” or “no”) to get smoking cessation counseling support; and (d) use of NRT products (“yes” or “no”), such as patch, gum, or lozenge during the study period. In addition, to complement our treatment fidelity monitoring of the telephone counselors [22], study participants rated the frequency (1 = “never”, 2 = “rarely”, 3 = “sometimes”, and 4 = “often”) with which the interventionist provided education, skill-building, and support related to reducing child TSE and quitting smoking.

### 2.7. Data Analytic Approach

First, we examined distributions for normality and outliers. Cotinine was not normally distributed, so we log-transformed it. Next, we examined patterns of missing data. We then used multiple imputation with chained equations to generate 40 multiply-imputed datasets and checked the convergence diagnostics and descriptive statistics across the imputations using the R package *mice* [48]. The imputation model included all of the variables in the analysis models plus the gender of both child and parent, race, marital status, education, income, parent age, age started smoking, whether or not there were household smoking rules, life stress, weight control, NRT use, smoking urges, smoking self-efficacy, smoke-free home self-efficacy, average cigarettes per day, total secondhand smoke exposure, self-reported quit status, and indicators for the hospital clinic. The analysis models for child TSE and parent quit status included the treatment condition as well as parents’ nicotine dependence level, depressive symptoms, child age, and whether or not there were other smokers besides the participant in the household. Even though we included many variables that were potentially predictive of missingness in the imputation model, we conducted sensitivity analysis to the missing at random assumption by assuming the worst-case scenario that everyone who was missing at the 12-month follow-up was smoking [49]. The results did not change from those reported below using the imputed data. The results from the 40 multiple-imputed datasets were combined using Rubin’s rules [50,51].

The inferential analyses used an intention-to-treat approach. We fit a linear mixed-effects model using the lmer function in the R package *lme4* [52] to examine the differences in log-cotinine between groups and across time (baseline to 12-month follow-up). In addition to the above-mentioned analysis model covariates, we included an interaction between treatment condition and time (in months) and a random intercept. We fit a logistic regression model to the 12-month home visit bioverified quit status, including the above-mentioned analysis model covariates. We used an alpha level of 0.05 for all of the statistical tests.

## 3. Results

### 3.1. Participant Enrollment, Retention, and Baseline Characteristics

**Figure 1:**
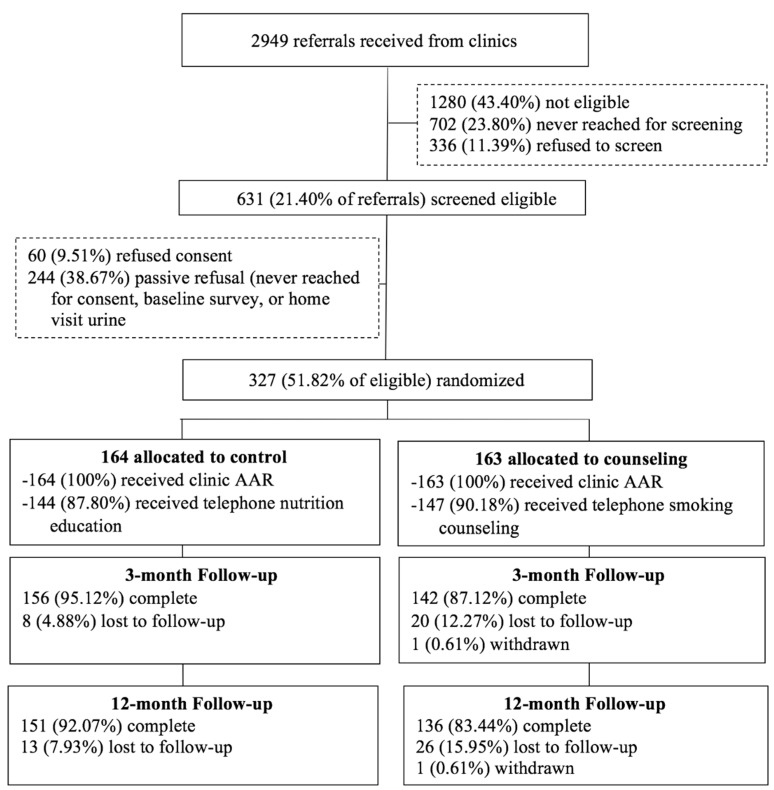
Participant flow chart.

Figure 1 shows the participant flow. We were able to stop recruitment with a final sample size of 327 (*n* = 163 AAR + counseling; *n* = 164 AAR + control), because 12-month attrition (12% of total sample) was below original projections. This sample size assures a power ≥0.80 to detect R^2^ change ranging from 0.03 to 0.05 (moderate effect size). Adjusting for clustering, there was no differential between-group attrition.

Table 1 shows descriptive data on the baseline sample characteristics, among which there were no between-group differences. The majority of parents were single, female, African American, lived below the national poverty level, and completed at least a high-school level education. About half of the sample reported significant depressive symptoms. Parents had a moderate smoking habit, at a little over a half-pack per day, and about half lived with another smoker. The sample of target children had a median age of five years, and approximately half were female.

### 3.2. Children’s Tobacco Smoke Exposure (TSE)

**Figure 2:**
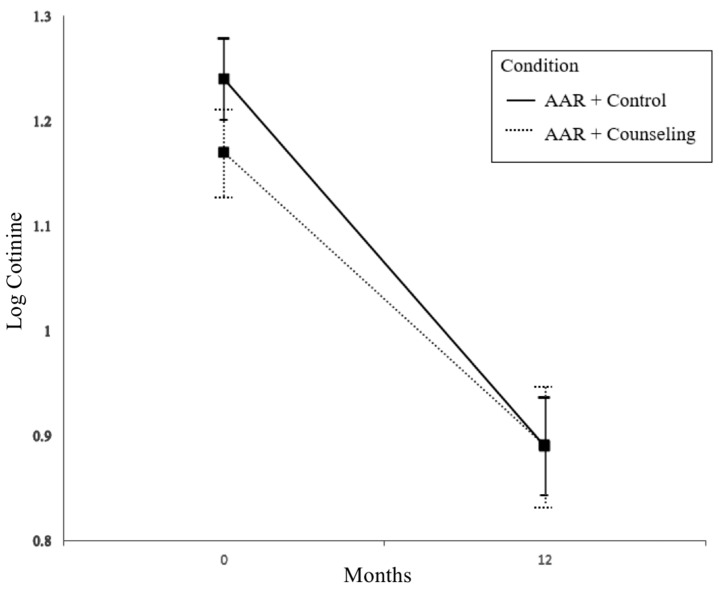
Change in log-cotinine from baseline to 12-month follow-up.

Neither the condition main effect nor the condition-by-months interaction was statistically significant (b = −0.079 and b = 0.007, respectively). The effect of time (in months) was statistically significant (b = −0.029, *t* (5476.893) = −6.741, *p* < 0.001), in that child TSE in both groups decreased between the baseline and 12-month follow-up time points (see Figure 2). The child age and parent nicotine dependence covariates had statistically significant effects (b = −0.002, *t* (3549.578) = −2.569, *p* = 0.01 and b = 0.047, *t* (6784.82) = 3.417, *p* < 0.001, respectively) on child log-cotinine, indicating higher TSE among children who were younger and children who had a parent with higher levels of nicotine dependence. Neither level of depressive symptoms nor the presence of other smokers in the household had a statistically significant effect on child log-cotinine (b = 0.000 and b = 0.098, respectively).

### 3.3. Parent Quit Status

At the 12-month follow-up, 15.2% were verified as having quit in the multilevel AAR + counseling intervention group versus 6.7% in the AAR + control group. Intention-to-treat analysis showed a statistically significant effect of the intervention on 12-month bioverified quit status (b = 0.903, *t* (218.43) = 2.199, *p* = 0.029), such that those in the intervention group were 2.47 (CI: 1.10, 5.54) times more likely to quit smoking than those in the control group. Parent nicotine dependence level had a statistically significant effect on bioverified quit status (b = −0.298, *t* (244.55) = −2.789, *p* = 0.006), indicating that more dependent parents were less likely to quit. However, the other covariates (i.e., child age, depressive symptoms, and other smokers in the household) did not have a statistically significant effect on 12-month bioverified quit status (b’s = 0.005, 0.006, and 0.244, respectively).

### 3.4. Parents’ Treatment Adherence

All of the participants received clinic-level AAR intervention, and a nearly equivalent percentage of participants received their randomly allocated telephone intervention in the AAR + counseling group (90.2%) and the AAR + control group (87.8%) (see Figure 1). On average, the multilevel AAR + counseling group participants achieved the adherence goal of completing ≥ three phone sessions (*M* = 3.35 ± 1.81). Table 2 shows the participants’ uptake of the recommendations that were delivered to all of the participants during the clinic phase of the intervention. These results show that by 12 months, significantly more participants in the multilevel AAR + counseling condition had used NRT than participants in the control condition (*p* < 0.004). Of those who used NRT during the study, few (4.4%) obtained a prescription from the pediatric provider. More often, NRT-using participants got a prescription from their personal physician (46.0%), paid out of pocket for over-the-counter NRT (20.4%), and received free NRT from the state quitline (18.6%), or received it from a friend or family member (10.6%). Table 2 also shows that the large majority of participants in both conditions took steps to protect children from TSE by implementing household and car smoking restrictions (>85%) and limiting children’s exposure to their own smoking (~two cigarettes per day) and from all of the sources, including themselves (~three cigarettes per day). Details on counseling process outcomes, such as self-efficacy and the use of urge-coping strategies, can be found in our earlier publication [22].

### 3.5. Interventionist Behaviors

As shown in Table 3, participants perceived that the telephone counselor frequently provided support, encouragement, and advice related to parents’ child TSE protection and cessation efforts. The median score for all of the items was four (“often”), which is the top of the scale.

## 4. Discussion

Children in the clinic-only, AAR + attention control intervention, which is the recommended standard of care in pediatrics, as well as those in the multilevel, AAR + counseling intervention, evidenced significant reductions in child TSE exposures as expressed in urine cotinine levels from baseline through to 12-month follow-up. Contrary to our hypothesis, our results suggest that the multilevel intervention did not further enhance reductions in cotinine compared with the control intervention. However, the multilevel intervention, which augmented the clinic AAR intervention with individualized, proactive telephone counseling for child TSE reduction and parental cessation, did improve bioverified smoking abstinence at the 12-month follow-up relative to the AAR + control condition: participants in the multilevel AAR + counseling condition were nearly two-and-a-half times more likely to quit than the clinic-only AAR + control participants. This outcome is remarkable, given that the target sample is known to face substantial hurdles in treatment access and engagement compared with the general population of smokers. Coinciding with the higher quit rate, participants in the multilevel KiSS condition were also significantly more likely to use NRT than control participants (56.6% versus 39.7%). There was evidence that participants’ behaviors in both conditions may have accounted for the overall reduced child cotinine: across conditions, there was evidence of increased restrictions on smoking in the home and the car, as well as a reduction in reported smoking around the child from baseline to 12-month follow-up (see Table 1 and Table 2). Therefore, our findings suggest that the clinic AAR intervention alone may be sufficient to promote reductions in TSE among the children of smoking parents, but a more intensive multilevel intervention is needed in order to promote successful smoking cessation among these parents.

The significant decline in cotinine levels in both groups is a positive outcome, but one that is difficult to interpret because of the lack of between-group differences. In a systematic review of child TSE intervention studies, 32 of the 57 reviewed studies showed reduced child TSE using various measures. For example, in a randomized trial comparing a usual care control group to a telephone counseling intervention designed to reduce child TSE and help parents quit smoking, child urine cotinine declined significantly over time in both groups [53]. One explanation is that the process of monitoring through repeated measures increased parents’ motivation to protect their child from TSE, which resulted in declines in exposure. Without additional control groups with no intervention and just assessment, we cannot estimate the extent to which reactivity to monitoring or assessments influenced outcomes.

It should be noted that study participants were made aware that the trial monitored child TSE (i.e., procedures to collect child urine to determine TSE) during the consent process, and again during the telephone data-collection phase that preceded home visit assessments at each time point. On average, one to two weeks passed between these notifications of monitoring and the collection of child urine at baseline. If parents were reacting to monitoring, they could have protected the child from exposures in advance of the first assessment, which would have generated low scores in baseline cotinine and not just at 12 months. A one-year delay in reactivity to monitoring does not seem plausible. Perhaps a more likely explanation of the cotinine findings is that the clinic AAR intervention implemented by a pediatric provider and driven by an EHR-guided decision aid, or an interaction of cotinine monitoring and the clinic AAR intervention, promoted protective behaviors and reduced child TSE. The convergence between parent-reported adherence to recommended child TSE protection behaviors and reduced cotinine over time suggest that the results are not simply an artifact of reactivity to measurement.

Despite significant reductions in child TSE indicated by cotinine changes over one year, and the general adoption of residential and car smoking restrictions among most of the study participants, child TSE was not eliminated. Previous research has shown that even when the children of smoking parents live in a home with smoking restrictions, their exposure to toxins from environmental tobacco smoke is still five to 10 times higher than that of the children of non-smoking parents [54]. Residual environmental tobacco smoke on household and car surfaces lingers and can be inhaled, ingested, or absorbed, particularly by young children who spend a significant amount of time in the home crawling around various surfaces and engaging in hand-to-mouth behavior [53]. One study found that environmental tobacco smoke contamination, as measured by nicotine in household dust, indoor air, and household surfaces, and child TSE, as measured by infant urine cotinine, were five to seven times higher in households with smokers who protected their children by smoking outside than in the households of non-smokers [7]. Smoking outside does not eliminate existing household contamination; it fails if doors or windows are opened near a smoker, and does not remove contamination from the skin, hair, and clothing of the smoker. Therefore, parents’ smoking cessation may be necessary in order to provide greater reduction in child TSE than just “taking it outside”. It may also be necessary to decontaminate environments once they are inhabited by smokers [7].

An important finding was the positive long-term effect of the intervention on bioverified smoking abstinence. Participants in both groups received three months of intervention followed by nine months of no treatment contact prior to the 12-month follow-up. To our knowledge, this is the first behavioral intervention for parent smokers that has demonstrated such long-term, bioverified smoking cessation effects. Moreover, this effect was observed in comparison to an active standard care intervention. Relatively few trials addressing child TSE and parent smoking have included either bioverified outcomes or post-treatment follow-up TSE and quit rates. A meta-analysis of five trials that included bioverified quit rates did not favor the intervention group, suggesting that the intervention group participants were not more likely to quit [55]. However, there was significant heterogeneity. In comparison with controls, two trials showed higher bioverified quit rates in the experimental group [40,56], whereas three trials showed no difference [57,58,59]. In the three trials with negative results, two emphasized TSE reduction more than smoking cessation as the target outcome. For example, in one of the negative trials, the intervention to reduce child TSE targeted mothers, but the majority (72%) of those mothers were not the smokers in the household [57]. In the other trial, the authors stated that “formal smoking cessation counseling” was not offered to participants, and they raised concerns about treatment fidelity (i.e., clinic staff were unable to reliably deliver the intervention according to protocol) [59]. The third negative trial was a pilot feasibility study that addressed cessation primarily through referral to a state quitline [58]. The pilot study had 48 participants in the intervention arm, but only 16 (33%) received telephone contact, which may have diluted treatment effects. Among the two trials showing positive effects on bioverified quitting, one emphasized parent cessation as the primary outcome [56], and the other emphasized child TSE reduction as a primary outcome [40]. Both trials showed short-term benefit, with the experimental participants having higher bioverified quit rates than controls at six months (15.3% versus 7.4%) [56] and four months (13.8% versus 1.9%) [40]. Together, the results of the current trial and prior research suggest that it is important to directly provide intervention to smokers, as opposed to other household members, and provide appropriately intensive smoking cessation elements in order to both protect the child from TSE and promote parent cessation.

In addition to the KiSS intervention providing substantial health education, support, and skills training for smoking cessation, and encouraging NRT usage, it may have been delivered to a highly-motivated population, even if they were not seeking smoking cessation treatment at the point of referral. As shown in Table 1, the vast majority (~80%) had made an attempt to quit for three or more days in the past. In addition, despite having smoked for many years, the average level of dependence was not high among participants. Nevertheless, our results suggest that low-income smokers engage in and respond favorably to intensive behavioral intervention delivered in a multilevel, pediatric-based framework, and that greater intervention intensity promotes higher quit rates than the standard approach. Since low-income smokers experience more challenges to quitting smoking and demonstrate lower quit rates in standard treatments than the general population of smokers, our results point to the need to disseminate more intensive interventions to address these disparities. However, the absolute percent who had quit at 12 months in our sample shows that there remains much room for improvement in future interventions targeting this population. As noted above, abstinence is critical for reducing children’s TSE. Parental smoking cessation is also critical for reducing tobacco-related morbidity and mortality among adults, and preventing children from becoming smokers themselves as they mature.

Toward the ongoing goal of improving intervention cessation rates, the KiSS intervention potentially could be improved by providing better access to NRT, more NRT choices, and opportunity for multiple NRT use. Greater than half of the multilevel KiSS participants used NRT, but many did not. Moreover, at EOT, we learned that many did not use NRT at effective doses [22]. Anecdotally, some participants mentioned that they disliked the taste of oral NRT, or they forgot to keep a supply of it on them. Such participants may prefer and benefit from a longer-acting patch rather than oral NRT. Direct provision of preferred NRT might also increase uptake. Additionally, incentives could be used to further improve intervention uptake and engagement in the behavioral intervention. Most participants received three or more telephone calls, which is greater contact time than standard quitlines [39]. However, the intervention might have been more effective in promoting cessation if more participants received all five calls. As a result of living in impoverished and often stressful environments, many participants had difficulty remembering or otherwise adhering to scheduled appointment times due to telephone service disruptions. Providing incentives might increase engagement and reduce barriers to participation.

Two non-program factors also predicted smoking cessation and TSE reduction, independent of intervention condition. Higher nicotine dependence in parents was associated with greater child TSE and a lower likelihood of quitting at 12-month follow-up. In future interventions, highly dependent smokers may be good candidates for more intensive pharmacological intervention to address withdrawal symptoms more effectively [35]. Many of the participants in this study had relatively low scores on the FTND, suggesting that a tailored or stepped intervention approach is appropriate to meet the diverse needs of this population. Younger children exhibited higher TSE than older children, which is consistent with previous research, but nonetheless disconcerting because they are more vulnerable to the adverse health effects of TSE [60]. Increasing efforts to help parents of infants and young children quit smoking and stay off tobacco may be an effective path for reducing TSE in this vulnerable population [9,61].

Depressive symptoms and the presence of other smokers in the home did not relate to outcomes in the multivariable models, suggesting that these are not major barriers to implementing the KiSS intervention. It is possible that other smokers in the home were generally agreeable about not smoking around children, as suggested by the overall declines in cotinine over time and in parents’ reports that relatively few cigarettes were being smoked near the child by them or others at 12-month follow-up. Other intervention studies with predominantly or all African American populations have shown mixed results with respect to the relation between depressive symptoms and smoking cessation. For example, one study found that baseline depressive symptoms did not predict abstinence in an African American sample participating in a randomized trial of bupropion effectiveness [62]. Another study found that baseline depressive symptoms were associated with poorer abstinence in a predominantly African American sample participating in a randomized trial of a behavioral activation smoking cessation treatment. Differences in study methodology may have contributed to the mixed results.

### Limitations

One limitation of this trial is the lack of a no-treatment control group. The clinic-level AAR intervention was designed to mirror the standard of care informed by guidelines for pediatric clinical practice for addressing parents’ smoking. However, using an active, standard care control group as the only comparison group to the multilevel KiSS intervention limits the interpretation of the declines in cotinine across conditions. Specifically, it is difficult to determine if the reduced child TSE can be attributed to the efficacy of the clinic intervention received by all of the participants or to an artifact, such as reactivity to monitoring children’s exposure. This limitation is not unique to our trial, suggesting that future trials should include relevant comparison groups to rule out artifacts to the extent that it is feasible. Another limitation is that we do not have data on the clinic implementation beyond participants’ observations. The use of the EHR certainly resulted in a high number of referrals within a short period of time. However, important details about the quality of the implementation are missing, such as how much time clinicians spent discussing the harms of child TSE, the benefits of protecting children from TSE, and the importance of and resources for quitting. Further, we have no data from clinicians about their attitudes toward the intervention (e.g., enthusiasm, burden, efficacy expectations). In addition, it is possible that applying the AAR approach consistently over a longer period of time might have yielded enhanced parental cessation. Future research would benefit from more attention to clinic implementation processes, sustainability, and outcomes to inform dissemination efforts and intervention improvements. Finally, the relatively low enrollment response rate combined with the exclusion of younger smokers, infrequent smokers, pregnant smokers, and those who may have mental health or other behavioral health problems limits the generalizability of the findings. A future pragmatic trial with fewer restrictions would be important for demonstrating efficacy in a broader population.

## 5. Conclusions

This research extends prior work on parental smoking cessation and child TSE reduction, which has shown mixed results on cessation and TSE reduction, particularly when minimal interventions and bioverified outcomes are used [13,14,55,63]. This trial showed that it is feasible to engage low-income adult smokers in an intensive TSE reduction and cessation intervention that is framed in a multilevel context of a pediatric clinic visit and telephone counseling. While the enrollment response rate was modest, some of that may be attributed to the burdens associated with the clinical trial (e.g., biomarker collection, home visits, lengthy surveys). In practice, this barrier would not exist, and individuals could be directly linked to a similar telephone-based intervention by pediatric health care providers. These findings are important because some pediatric health care providers are reticent to address parental smoking, citing barriers such as a lack of time, low confidence in effectiveness, and concern about damaging the therapeutic relationship with the parent [64,65]. Recent evidence suggests that parental tobacco control interventions are also scalable and sustainable in pediatric practice [66].

The multilevel KiSS intervention combining AAR with telephone counseling quickly generated hundreds of referrals and produced positive effects on long-term smoking abstinence in a low-income, distressed sample of adult smokers. There is also suggestive evidence that the clinic-level AAR intervention alone may have promoted parent protective behaviors and reduced child TSE. The multilevel intervention could be adopted by pediatric clinics, given the widespread availability of EHR systems that can be programmed to give prompts to health care providers to engage in brief AAR and even link smoking parents to freely available state quitlines using electronic referral systems [67]. For the program to be most effective, the quitlines should be proactive and incorporate TSE reduction education in addition to cessation counseling and support, as in the KiSS intervention. Some state quitlines already provide NRT, which would be an added strength. Delivering intervention to adults at pediatric office visits is an effective strategy for addressing the excess burden of child TSE in low-income and minority communities.

## Figures and Tables

**Table 1:** Baseline participant characteristics by condition (*n* = 327).

Characteristic	AAR ^1^ + Control (*n* = 164)	AAR + Counseling (*n* = 163)	*p*-Value
Parent age, mean ± SD, years	33.9 ± 9.2	32.7 ± 7.9	NS ^2^
Parent female gender, %	81.1	85.9	NS
Parent African American race, %	84.1	82.2	NS
Parent married/living with partner, %	38.4	43.6	NS
Parent education less than high school, %	28.0	26.4	NS
Parent income below poverty level, %	78.7	78.5	NS
Parent significant depressive symptoms ^3^, %	50.0	52.1	NS
Other smokers in home, % yes	47.0	45.4	NS
Smoking restrictions in home/car, % yes	70.7	68.7	NS
Cigarettes smoked daily near child, all sources, mean ± SD	7.7 ± 7.8	8.6 ± 9.6	NS
Cigarettes smoked per day near child by parent, mean ± SD	5.6 ± 4.9	5.9 ± 5.2	NS
Parent previously quit for at least 3 days in a row, % yes	79.9	81.0	NS
Parent nicotine dependence ^4^, mean ± SD	4.0 ± 1.9	4.2 ± 2.0	NS
Parent cigarettes smoked per day, mean ± SD	10.7 ± 5.8	12.2 ± 8.9	NS
Parent age started smoking, mean ± SD, years	17.4 ± 3.5	17.4 ± 4.3	NS
Child age, mean ± SD, months	64.0 ± 33.9	64.5 ± 31.6	NS
Child female gender, %	47.0	51.5	NS
Cotinine (log), mean ± SD	1.24 ± 0.50	1.17 ± 0.53	NS

Notes: ^1^ AAR = “Ask, Advise, Refer”; ^2^ NS = not significant at *p* < 0.05, two-tailed; ^3^ Possible range 0 to 30, higher scores equal more depressive symptoms, and significant depressive symptoms defined as a score greater than or equal to 10 on the depressive symptoms measure; ^4^ Possible range 0 to 10, higher scores equal more dependence.

**Table 2:** Parent adoption of smoking cessation and protective behaviors at 12-month follow-up by condition (*n* = 287).

Behavior	AAR ^1^ + Control (*n* = 151)	AAR + Counseling (*n* = 136)	*p*-Value
Smoking restrictions in home/car, % yes	87.4	85.9	NS ^2^
Cigarettes smoked daily near child, all sources, mean ± SD	3.04 ± 3.84	3.80 ± 5.52	NS
Cigarettes smoked per day near child by parent, mean ± SD	2.01 ± 2.85	1.98 ± 2.61	NS
Contacted state quitline during study period %	27.8	25.7	NS
Used NRT ^3^ during study period, %	39.7	56.6	0.004

Notes: ^1^ AAR = “Ask, Advise, Refer”; ^2^ NS = not significant at *p* < 0.05, two-tailed. ^3^ NRT = nicotine replacement therapy.

**Table 3:** Parents in Ask, Advise, Refer + Counseling condition ratings of phone counselor behaviors at the 12-month follow-up (*n* = 163).

Behavior	Mean ± SD ^1^
Expressed confidence in my ability to create a smokefree home	3.77 ± 0.60
Expressed pleasure at my efforts to protect my child from tobacco smoke	3.79 ± 0.57
Acted supportive of my efforts to protect my child from tobacco smoke	3.85 ± 0.48
Offered helpful tips for keeping my child away from tobacco smoke	3.85 ± 0.48
Complimented me on my efforts to not smoke	3.70 ± 0.70
Helped me think of substitutes for smoking	3.73 ± 0.66
Expressed confidence in my ability to quit or continue to abstain from smoking	3.79 ± 0.55
Encouraged me to use substitutes for cigarettes	3.66 ± 0.72
Suggested activities that I could do to keep from smoking, such as go for a walk	3.77 ± 0.60
Encouraged me to get nicotine replacement, such as gum or the patch	3.59 ± 0.84

Note: ^1^ Scale range from 1 = “never” to 4 = “often”.
